# Supplementary figures and images for: Muscle overexpression of Klf15 via an AAV8-Spc5-12 construct does not provide benefits in spinal muscular atrophy mice
Source: Gene Ther. 2020 Apr 20;27(10):505–15. doi: 10.1038/s41434-020-0146-8 (PMC7674152; doi:10.1038/s41434-020-0146-8)

\* [ *Smn*<sup>-/-</sup>;SMN2 (AAV8-*Klf15* 1E10 vg/pup)  
*Smn*<sup>-/-</sup>;SMN2 (AAV8-*Klf15* 2E10 vg/pup)

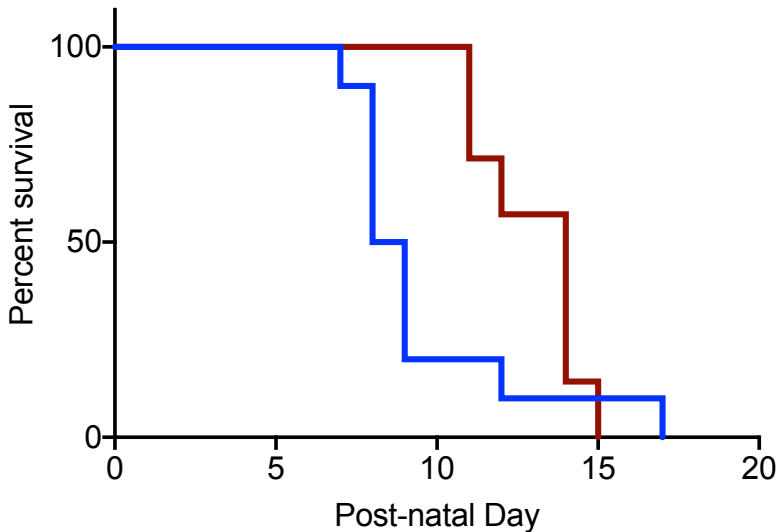

Supplement: Supplementary file 1 — Supplementary Figure 1 [file 41434_2020_146_MOESM1_ESM.pdf]

Relative expression compared to Quad  
(expression normalized to *PolJ*)

P2

P7

- Quad
- Heart
- ▲ Liver

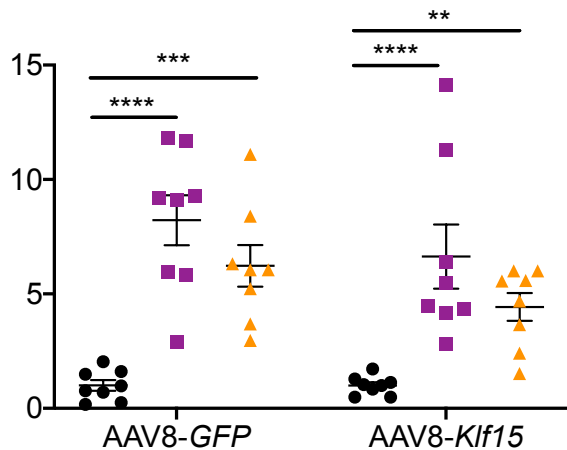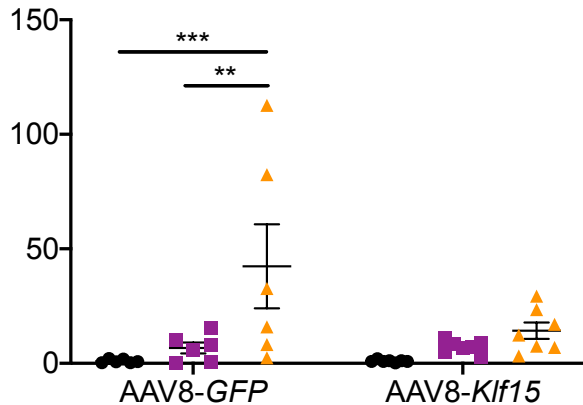

Supplement: Supplementary file 2 — Supplementary Figure 2 [file 41434_2020_146_MOESM2_ESM.pdf]
