# Supplementary material for: Muscle overexpression of Klf15 via an AAV8-Spc5-12 construct does not provide benefits in spinal muscular atrophy mice
Source: Gene Ther. 2020 Apr 20;27(10):505–15. doi: 10.1038/s41434-020-0146-8 (PMC7674152; doi:10.1038/s41434-020-0146-8)
Supplement: Supplementary file 3 — Supplementary Figure Legends [file 41434_2020_146_MOESM3_ESM.pdf]

SUPPLEMENTARY FIGURE LEGENDS

Supplementary FIG. 1. An AAV-*Klf15* dose of 1E10 vg/pup does not provide additional survival benefits compared to a dose of 2E10 vg/pup. Survival curves of *Smn*$^{-/-}$;*SMN2* SMA mice that received a single facial vein intravenous injection at post-natal day (P) 0 of 1E10 vg/pup of AAV8-*Klf15* (n = 10) or 2E10 vg/pup of AAV8-Klf15 (n = 7). Data are Kaplan-Meier survival curves, Gehan-Breslow-Wilcoxon test, *p = 0.0113.

Supplementary FIG. 2. AAV8-Spc-12 tropism is greater in heart and liver of neonatal pups. Post-natal day (P) 0 *Smn*$^{-/-}$;*SMN2* SMA mice and control littermates received a single facial vein intravenous injection of AAV8-*GFP* or AAV8-*Klf15* (2E10 vg/pup). qPCR analysis of *AAV8* expression in quadriceps, heart and liver of of P2 and P7 AAV8-*GFP* and AAV8-*Klf15*-treated mice. Data are scatter plot and mean ± SEM, n = 6–8 animals per experimental group, two-way ANOVA, **p<0.01, ***p<0.001, ****p<0.0001.
